# Distal Clavicle Excision: An Epidemiologic Study Using the National Ambulatory Surgery Sample Database

**DOI:** 10.7759/cureus.22092

**Published:** 2022-02-10

**Authors:** Joshua D Meade, Bradley L Young, Ziqing Yu, David P Trofa, Dana P Piasecki, Nady Hamid, Shadley Schiffern, Bryan M Saltzman

**Affiliations:** 1 Orthopedics, Musculoskeletal Institute, Atrium Health, Charlotte, USA; 2 Orthopedic Surgery, Musculoskeletal Institute, Carolinas Medical Center, Atrium Health, Charlotte, USA; 3 Orthopedic Surgery, Atrium Health, Charlotte, USA; 4 Orthopedic Surgery, Columbia University Irving Medical Center, New York, USA; 5 Sports Medicine Center, OrthoCarolina, Charlotte, USA; 6 Sports Medicine center, OrthoCarolina, Charlotte, USA

**Keywords:** national trends, nass database, arthroscopy, rotator cuff repair, distal clavicle excision

## Abstract

Background: This study aimed to examine national trends pertaining to patient demographics and hospital characteristics among distal clavicle excision (DCE) procedures performed in the United States.

Methods: The National Ambulatory Surgery Sample (NASS) database was queried for data. Encounters with Current Procedural Terminology (CPT) code 29824 were selected. Metrics derived from these encounters included patient demographic information such as age, geographic location, median household income per zip code, and primary expected insurance payer. Hospital characteristics derived included total charges for DCE procedures, location of the hospital, disposition of the patient, hospital census region, control/ownership of the hospital, and location/teaching status of the hospital. The proportion of DCE performed concomitantly with rotator cuff repair (RCR) was also analyzed. P-values were obtained from continuous variables using a t-test with a linear regression model. P-values were obtained from event variables using chi-square analysis.

Results: The incidence of arthroscopic DCE in the US decreased from 99,070 in 2016 to 93,678 (5.5%) in 2018. Of note, the proportion of DCE performed concomitantly with RCR significantly increased from 50.4% in 2016 to 52.8% in 2018 (P < 0.0001). Median patient age increased from 2016 to 2018 (56.4 to 57.2; P* *< 0.0001). The income quartile that saw the highest number of encounters was between $43,000 and $53,999 (P* *< 0.0001). Hospital trends display an increasing cost from $16,944 to $18,855 over the study period (P* *= 0.0016). Private insurance, including health maintenance organizations (HMOs), were the largest payers for this procedure; however, a decreasing trend in DCE covered by private insurance was noticed (50.2% to 47.3%; P < 0.0001). Medicare was the second-largest payer ranging from 27.9% in 2016 to 29.9% in 2018. The urban teaching model of hospitals continues to see the highest number of encounters for this procedure.

Conclusions: In both 2016 and 2018, private insurance was the most common payer, most DCEs were performed in urban teaching hospitals, and most patients undergoing the procedure had a median household income between $43,000 and $59,000. Between 2016 and 2018, there was a significant increase in costs associated with DCE, as well as an increase in the median age of patients undergoing the procedure. The proportion of DCE performed concurrently with RCR also significantly increased during the study period.

## Introduction

Distal clavicle excision (DCE) was originally described in 1941 by Mumford and Gurd [1]. DCE is commonly performed in the surgical management of acromioclavicular (AC) joint osteoarthritis. A variety of modalities are used to diagnose AC joint pathology, with radiographic imaging being the gold standard [2]. This diagnosis can also be made clinically as patients may have radiographic signs of AC joint osteoarthritis without any symptoms. Plain radiographic films may show narrowing of the joint space with or without osteophytes, sclerotic changes, and periarticular cysts. The technetium-99 bone scan can also be utilized to look for areas of uptake that may highlight osteolysis in the distal clavicle; however, this more advanced imaging technique is rarely used due to the high sensitivity of clinical and radiographic studies [3].

DCE can be performed via the classical open technique or arthroscopically [3]. The incidence of open DCE has decreased in lieu of the increasing popularity of arthroscopic techniques. A recent study by Forlenza et al. showed that the annual rate of arthroscopic DCE in the United States increased from 53.9% to 69.8% between 2007 and 2016 [4]. Arthroscopic DCE has also been shown to have more favorable outcomes with regards to postoperative pain, faster return to activities of daily living, and improved 36-Item Short Form Survey (SF-36) scores [5]. Arthroscopic DCE has been shown to have a similar reimbursement rate to the open procedure, with little to no difference being reported in total operating time [6].

While literature exists pertaining to clinical outcomes, complication rates, and other facets of DCE, there is little epidemiologic data concerning patient demographics or hospital characteristics where these procedures are performed. This epidemiologic study examined patient demographics as well as various hospital characteristics in an effort to evaluate emerging trends. This study aimed to provide surgeons, hospitals, insurance companies, and policymakers with information surrounding patient demographics and hospital characteristics in the setting of DCE.

## Materials and methods

The National Ambulatory Surgery Sample (NASS) database was queried for patients who underwent arthroscopic excision of the distal clavicle using Current Procedural Terminology (CPT) code 29824. The NASS is part of the Healthcare Cost and Utilization Project (H-CUP). This database contains information from a variety of hospitals and major ambulatory surgery centers pertaining to patient demographics, source of payments, type of surgical procedure, as well as insurance information. The NASS is the largest known surgery database in the United States, accounting for over 7.7 million ambulatory surgery encounters annually.

Certain metrics were derived from the dataset of patients who underwent DCE, including gender, age, geographical location of patients’ home address, median household income, and primary expected payer. Hospital characteristics that were derived included total hospital charges, concurrent rotator cuff repair (RCR) performed with DCE, location of hospital (rural or urban), hospital census region, ownership of hospital (voluntary, public, and proprietary), patient disposition (routine, transfer to short-term hospital, home healthcare, leave against medical advice, others, and missing), and teaching status of the hospital. P-values for the continuous variables such as age and total charges were derived from a t-test with a linear regression model. P-values for the event values such as the location of the hospital or disposition of the patient were obtained from the chi-square analysis. A significance value of less than 0.05 was set for P-values after undergoing trend analysis.

## Results

Based on the NASS database, the incidence of arthroscopic DCE in the US decreased from 99,070 in 2016 to 93,678 (5.5%, P = 0.22) in 2018 (Table 1).

**Table 1:** National estimates of encounters.

	N = 290,838
2016	99,070
2017	98,090
2018	93,678

However, the proportion of DCE performed concomitantly with RCR increased from 50.4% in 2016 to 52.8% in 2018 (P < 0.0001). Additionally, the median age of encounters has increased from 2016 to 2018 (56.4 to 57.2; P < 0.0001). There was also a difference in which patients underwent DCE based on where they lived, with the majority of patients coming from a medium metropolitan location (defined as a county population containing between 250,000 and 999,999 individuals according to the CDC National Center for Health Statistics), and the lowest number of patients coming from neither metropolitan nor micropolitan locations (P < 0.0001). The largest proportion of DCEs was performed in patients who had a median household income between $43,000 and $53,999 (28.8% in 2016 to 31.3% in 2018 of total encounters; P < 0.0001). Per Table 2, the primary payer for DCE was largely private insurance, including health maintenance organizations (HMOs), with Medicare being the second-largest payer (P < 0.0001). The number of private insurance payers did decrease during the time period studied (50.2% to 47.3%; P < 0.0001).

**Table 2:** Patient characteristics by years. * P-values by trend analysis. HMO, health maintenance organization.

				P-value
	2016 (N = 99,070)	2017 (N = 98,090)	2018 (N = 93,678)	0.22
Sex				
Male	54,595 (55.1%)	54,469 (55.5%)	51,473 (54.9%)	0.44*
Female	44,433 (44.9%)	43,620 (44.5%)	42,205 (45.1%)	
Age at admission				
Median (95% CI)	56.4 (56.2, 56.6)	56.7 (56.5, 56.9)	57.2 (56.9, 57.5)	0.0001*
Patient location				
Large central metropolitan	17,704 (17.8%)	17,046 (17.3%)	15,571 (16.6%)	0.0001
Large fringe metropolitan	22,511 (22.7%)	21,752 (22.1%)	20,956 (22.3%)	
Medium metropolitan	22,172 (22.3%)	23,863 (24.3%)	22,838 (24.3%)	
Small metropolitan	10,046 (10.1%)	9,781 (9.9%)	9,101 (9.7%)	
Micropolitan	16,399 (16.5%)	15,483 (15.7%)	15,226 (16.2%)	
Not metropolitan or micropolitan	10,159 (10.2%)	10,113 (10.3%)	9,956 (10.6%)	
Median household income national quartile for patient ZIP code				
$1-42,999	23,626 (23.8%)	23,444 (23.9%)	22,317 (23.8%)	0.0001
$43,000-$53,999	28,564 (28.8%)	29,523 (30.1%)	29,287 (31.3%)	
$54,000-$70,999	25,463 (25.7%)	24,777 (25.3%)	23,334 (24.9%)	
$71,000 or more	19,481 (19.7%)	18,834 (19.2%)	17,455 (18.6%)	
Primary expected payer				
Medicare	27,707 (27.9%)	28,124 (28.7%)	28,078 (29.9%)	0.0001
Medicaid	8,054 (8.1%)	8,508 (8.7%)	8,076 (8.6%)	
Private including HMO	49,706 (50.2%)	48,191 (49.1%)	44,310 (47.3%)	
Self-pay	803 (0.8%)	750 (0.8%)	779 (0.8%)	
No charge	39 (0.04%)	48 (0.05%)	37 (0.04%)	
Other	12,621 (12.7%)	12,251 (12.5%)	12,231 (13.1%)	

Total median charges per ambulatory surgery encounter for DCE increased from $16,944 in 2016 to $18,855 in 2018 (P = 0.0016) (Table 3). This increase in total charges during the study period remained significant after adjusting for inflation. The locations of hospitals where the procedures were performed were largely urban settings (80.1% in 2016 to 80.4% in 2018). The proportion of patients who had a routine disposition (those who were discharged to home and self-care) after DCE increased over the study period (P < 0.0001) from 91.5% to 93.7%. The urban teaching model of hospitals was also the most utilized and continues to trend upward during the study period (49.4% to 52.7%; P < 0.0001), with the urban non-teaching model seeing the second-highest number of encounters (30.7% in 2016 to 27.7% in 2018).

**Table 3:** Hospital characteristics by years. * P-values by trend analysis. ** In 2018 dollars. *** Skilled nursing facility, intermediate care, and other types of facility.

	2016 (N = 99,070)	2017 (N = 98,090)	2018 (N = 93,678)	P-value
Total charges**				
Median (95% CI)	16,944 (15,805, 18,083)	17,646 (16,440, 18,850)	18,855 (17,420, 20,289)	0.0016*
Concurrent rotator cuff repair				
N (%)	49,974 (50.4%)	51,041 (52.0%)	49,432 (52.8%)	0.0001*
Location of the hospital				
Rural	19,698 (19.9%)	18,630 (19%)	18,379 (19.6%)	0.12*
Urban	79,371 (80.1%)	79,460 (81%)	75,299 (80.4%)	
Disposition of patient				
Routine	90,706 (91.5%)	90,238 (92%)	87,797 (93.7%)	0.0001
Transfer to short-term hospital	75 (0.1%)	65 (0.1%)	32 (0.03%)	
Home healthcare	209 (0.2%)	199 (0.2%)	248 (0.3%)	
Against medical advice	2 (0%)	12 (0.01%)	6 (0.01%)	
Other transfers***	108 (0.1%)	95 (0.1%)	118 (0.1%)	
Missing	7,968 (8%)	7,477 (7.6%)	5,457 (5.8%)	
Hospital census region				
Northeast	14,376 (14.5%)	14,656 (14.9%)	14,413 (15.4%)	0.0001
Midwest	28,145 (28.4%)	26,896 (27.4%)	25,670 (27.4%)	
South	39,695 (40.1%)	39,822 (40.6%)	38,314 (40.9%)	
West	16,853 (17%)	16,715 (17%)	15,279 (16.3%)	
Control/ownership of the hospital				
Public	10,940 (11%)	11,684 (11.9%)	12,098 (12.9%)	0.0001
Voluntary	78,067 (78.8%)	76,162 (77.6%)	73,399 (78.4%)	
Proprietary	10,061 (10.2%)	10,244 (10.4%)	8,180 (8.7%)	
Location/teaching status of the hospital				
Rural	19,698 (19.9%)	18,630 (19%)	18,379 (19.6%)	0.0001
Urban non-teaching	30,465 (30.7%)	28,166 (28.7%)	25,952 (27.7%)	
Urban teaching	48,905 (49.4%)	51,293 (52.3%)	49,346 (52.7%)	

## Discussion

Conservative management of AC joint pathology is generally recommended before surgical intervention [7]. However, if conservative management fails, open or arthroscopic DCE can be performed in appropriate candidates. Recent trends show an increase in the number of arthroscopic DCEs being performed compared to the open procedure [6,8,9]. This could be in part due to an increased complication rate in the open procedure vs. arthroscopic, as well as less pain after surgery in the arthroscopic subgroup [3,6,8,10,11]. The open DCE approach has also been shown to violate the superior and posterior AC ligaments more often than the arthroscopic approach [8,12]. Some common postoperative complications include persistent pain, incomplete resection, stiffness, and, less commonly, infection or fracture [11,13].

It is important to evaluate emerging trends in costs and insurance coverages for patients. Many hypotheses exist as to why the lower socioeconomic group of patients may undergo arthroscopic DCE more often than other socioeconomic groups. These include a more robust risk factor profile such as high psychosocial job demand, more repetitive shoulder movement, awkward posturing, and lack of exercise or poor diet [14-17]. Another recent epidemiologic study found that the highest incidence of DCE was in men, ages 55-64 years, and in the most socioeconomically deprived [15].

In the current state of health care, where cost is heavily scrutinized, arthroscopic shoulder surgery remains one of the most prevalent procedures performed [17]. This epidemiologic study reported on current trends in demographic information relating to the DCE using the NASS database. This study found that the total ambulatory surgery charges increased for the DCE procedure between 2016 and 2018, while private insurers, including HMOs, became less likely to be the primary payer during the study period. A variety of factors could point to why the charges are increasing for this procedure. With the increased incidence of these procedures being performed in urban teaching hospitals, it is possible that increased support staff in the operating room could lead to increased OR time and ultimately increased cost for this procedure. In contrast to previously published literature, this study found that the incidence of ambulatory, arthroscopic DCE in the US decreased between 2016 and 2018. Urban teaching hospitals continue to increase their numbers of encounters while rural hospitals continue to trend downward. Efforts to improve outpatient orthopedic procedures may be leading to the increase in the routine disposition of patients undergoing arthroscopic DCE.

When interpreting the data presented concerning total ambulatory charges for patients who underwent DCE, it is important to understand that DCE is often performed in conjunction with RCR, subacromial decompression, or biceps tenodesis. It is possible that concomitant surgeries could influence operative time and total charges reported in NASS. Specifically, a study by Curry et al. found that the male sex, beach chair positioning, and the number of tendons involved in the RCR all increased the length of the operation [10]. Another recent study by Iyengar et al. used a Florida surgical database and showed that from the year 2003 to 2007, there was a large increase in additional arthroscopic procedures performed concomitantly with RCR, including a 589% increase in DCE [18]. Our study lends support to the idea that longer procedure time with concurrent procedures being performed increases costs and total operating time for arthroscopic DCE.

Limitations

Despite this being the largest and most up-to-date evaluation of the current epidemiology of DCE in the US, there are inherent limitations to studies based on large, nationally representative databases. Given a large amount of data and the complexity of the new International Classification of Diseases, Tenth Revision (ICD-10) coding system used in NASS, human error during coding is always a possibility. Secondly, NASS only contains data from hospital-owned facilities. Therefore, care should be taken with extrapolating these data to physician-owned ambulatory surgery centers. Another limitation is that NASS includes data from 31 states. Policymakers and surgeons should make note of which states these data are coming from when they are interpreting these findings.

## Conclusions

In both 2016 and 2018, private insurance was the most common payer for DCE. Most DCEs were performed at urban teaching hospitals, and most patients undergoing DCE had a median household income of around $43,000 and $59,000. There was a significant increase in costs associated with DCE, as well as an increase in the median age of patients undergoing the procedure. A significant increase in DCE procedures was noted among patients undergoing concomitant RCR. Despite the apparent increase in costs and patient age over time, a larger proportion of patients undergoing DCE at ambulatory surgery centers are experiencing a routine discharge.
